# Medical Opinion and Movement

**Published:** 1907-03-02

**Authors:** 


### Medical Opinion and Movement.

The terrible havoc of disease that was wrought in our South African Forces owing to the utter lack of proper measures of sanitation and the ignorance displayed by the military authorities on the subject has been brought into vivid contrast with the experience of the Japanese Army in Manchuria. The War Office at last shows signs of an awakening intelligence in the matter, and evidently intends to profit by the lesson, and to include sanitation amongst the other measures of reorganisation which are making themselves manifest. The Army Orders for February make provision for instruction of the officers, and also of the non-commissioned officers and men, in sanitation. An official text-book on military sanitation is to be issued and annual courses of lectures are to be given. Special classes are to be given to officers at the Aldershot School of Army Sanitation, and other classes are to be arranged for regimental non-commissioned officers and men. An examination in sanitation, based upon the official text-book, will be included in the final examination of cadets in the senior division after March 1, 1908. Furthermore, on mobilisation a sanitary inspection committee is to be formed for service in the field. Its duties will be to superintend the provision of all sanitary appliances and materials, to assist officers and the Medical Service to maintain the health of the Army, to initiate schemes of general sanitation, to inspect stations occupied by troops, and to further in every way the maintenance of sanitary conditions.

The state of shock and the causal factors underlying it form a subject of considerable interest to the physiologist, and have also become one of practical importance to the surgeon. In recent years the matter has been very carefully studied by Dr. G. W. Crile, and his work and the conclusions he arrived at have been confirmed by Mr. J. P. Mummery and others. Briefly expressed, these authors hold the view that the condition of shock consists essentially in an exhausted state of the vaso-motor centres, giving rise to a general paralysis of the blood-vessels throughout the body and a consequent fall of blood-pressure. Mr. J. D. Malcolm raises the question anew, and in a paper read before the Royal Medical and Chirurgical Society contends that the essential factor involved in shock consists in a general constriction of the blood-vessels. He brings no new facts or observations in support of his theory, but is of opinion that it is a more proper interpretation of the facts already known. This opinion was not shared by any member of the Society who took part in the discussion, and is, we think, hardly likely to receive any general support. It is difficult to understand, for instance, his contention that a general constriction of the blood-vessels would give rise to a fall of blood-pressure. It is surely sufficiently well established by the laws of physics that any general constriction of the blood-vessels causes increased resistance in the flow of blood, which must be accompanied by increase of blood-pressure behind that resistance. Mr. Malcolm appears to lay much stress on the fact that in shock the peripheral vessels are empty; but, as Dr. A. P. Beddard pointed out, this is secondary to the fall of blood-pressure, and the accumulation of the blood in the large veins of the splanchnic area.

The question of compulsory vaccination gave rise to an animated debate in the House of Commons. Mr. Lupton moved an amendment to the address in favour of the abolition of the penal clauses of the Vaccination Acts, and elicited a promise from Mr. Burns that a Bill would be introduced by which it will be possible to obtain exemption from vaccination by a statutory declaration. The penal clauses have undoubtedly received varying interpretations in different parts of the country, and the conscientious objector has received somewhat harsh treatment at the hands of magistrates in certain districts, while in others compulsory vaccination has been in name only. The Bill now to be introduced will mete out the same treatment all over the country, but there is danger lest its effect will be to so simplify the way to exemption from vaccination as to annul the force of the original Act altogether. It might have been supposed that the overwhelming evidence which the profession has ready to hand in favour of yet more stringent measures for vaccination and re-vaccination of the community might have reached the intelligence of our legislators and helped them to form opinions on the subject in conformity with the facts. On the contrary, it is well known that there is a large contingent in the House in favour of the abolition of compulsory vaccination altogether. Their knowledge on the subject may be judged from their spokesman on this occasion, who referred to vaccination as "the most wicked invention of opening a vein, cutting through the skin, and putting into it pus from a diseased cow." Under the circumstances it seems probable that vaccination is doomed to receive a considerable set-back in this country until some serious outbreak of the disease again brings vividly before the mind of the community the horrors of the malady.

Cerebro-spinal fever shows no sign of abatement at the two centres, Glasgow and Belfast, where it is most prevalent. Fresh cases are reported daily, and the mortality is very high. Further outbreaks of the disease are reported in several Scotch and Irish towns, and during the past week there have been sporadic cases in different parts of the country and also in London. On the recommendation of the Public Health Committee, the London County Council has decided to make the disease notifiable in the administrative county of London for a period of six months. The Local Government Board has issued a circular to sanitary authorities on the subject, pointing out that should groups of cases of illness at all resembling in nature cerebro-spinal fever occur in any district, the fact is to be reported upon specially by the medical officer of health, and a copy of the report sent to the board. Accompanying the circular is a copy of the memorandum of the disease drawn up in 1905 by Mr. W. H. Power, Medical Officer to the Board.

